# Contributions from both the brain and the vascular network guide behavior in the colonial tunicate *Botryllus schlosseri*

**DOI:** 10.1242/jeb.244491

**Published:** 2022-11-25

**Authors:** Stuart H. Thompson, Chiara Anselmi, Katherine J. Ishizuka, Karla J. Palmeri, Ayelet Voskoboynik

**Affiliations:** ^1^ Department of Biology and Hopkins Marine Station, Stanford University, Stanford, CA 93950, USA; ^2^ Institute for Stem Cell Biology and Regenerative Medicine, Stanford University School of Medicine, Stanford, CA 94305, USA; ^3^ Chan Zuckerberg Biohub, San Francisco, CA 94158, USA

**Keywords:** Colonial tunicate, Neural activity, Excitable epithelium, Synchronous and autonomous behavior

## Abstract

We studied the function, development and aging of the adult nervous system in the colonial tunicate *Botryllus schlosseri.* Adults, termed zooids, are filter-feeding individuals. Sister zooids group together to form modules, and modules, in turn, are linked by a shared vascular network to form a well-integrated colony. Zooids undergo a weekly cycle of regression and renewal during which mature zooids are replaced by developing buds. The zooid brain matures and degenerates on this 7-day cycle. We used focal extracellular recording and video imaging to explore brain activity in the context of development and degeneration and to examine the contributions of the nervous system and vascular network to behavior. Recordings from the brain revealed complex firing patterns arising both spontaneously and in response to stimulation. Neural activity increases as the brain matures and declines thereafter. Motor behavior follows the identical time course. The behavior of each zooid is guided predominantly by its individual brain, but sister zooids can also exhibit synchronous motor behavior. The vascular network also generates action potentials that are largely independent of neural activity. In addition, the entire vascular network undergoes slow rhythmic contractions that appear to arise from processes endogenous to vascular epithelial cells. We found that neurons in the brain and cells of the vascular network both express multiple genes for voltage-gated Na^+^ and Ca^2+^ ion channels homologous (based on sequence) to mammalian ion channel genes.

## Teaser

**Highlighted Article:** In the colonial tunicate *Botryllus schlosseri*, both the nervous systems of zooids and the excitable vasculature contribute to generating behavior. Both tissues produce patterned output and both express voltage-dependent Na^+^ and Ca^2+^ ion channels.

## INTRODUCTION

The colonial tunicate *Botryllus schlosseri* is an invertebrate chordate, a member of a sister group to vertebrates (Urochordata; Delsuc et al., 2006). Its relationship to vertebrates and its compact and well-annotated genome (Voskoboynik et al., 2013; Delsuc et al., 2018) have made *B. schlosseri* an attractive model organism for studies of allorecognition, stem cell and developmental biology, aging, neurogenesis and neurodegeneration (Anselmi et al., 2022; Kowarsky et al., 2021; Laird et al., 2005; Manni et al., 2014; Rosental et al., 2018; Voskoboynik and Weissman, 2015; Voskoboynik et al., 2020 preprint). In this study, we took advantage of the properties of *B. schlosseri* colonies to gain insight into the neurobiology and behavior of a species at the base of the vertebrate evolutionary tree.

**Fig. 1.**
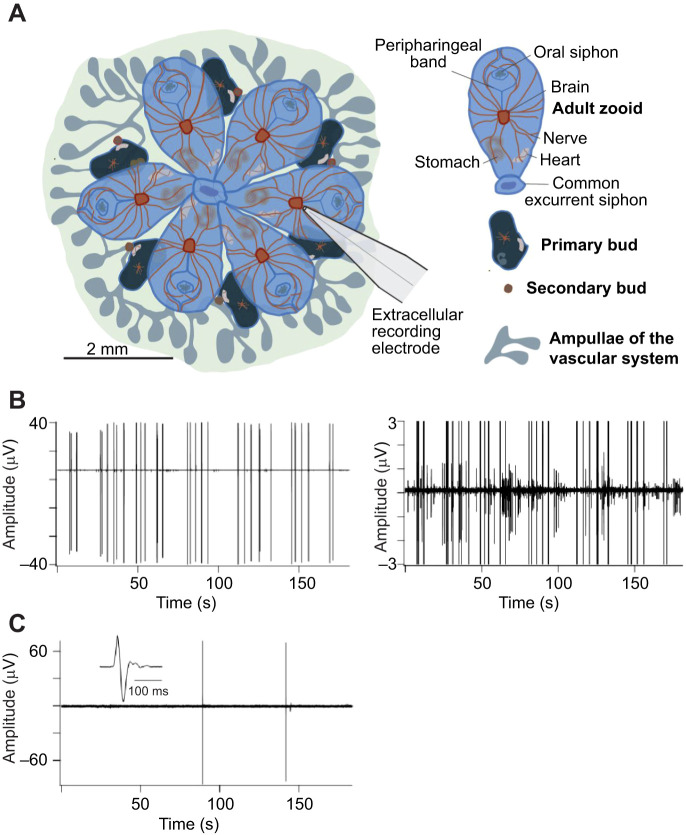
**Brain activity at early and late developmental stages.** (A) Illustration of a *Botryllus schlosseri* colony composed of a single module of six adult zooids with their associated primary and secondary buds, the location of the major organs, and the ampullae of the vascular network (credit: C. Anselmi). A recording electrode is shown positioned over the brain approximately to scale. (B) Extracellular recording from the brain of a mature zooid at developmental stage A3 showing spontaneous action potentials. The record is replotted at higher amplification to the right to emphasize the diversity of action potential waveforms. The amplitudes of the largest action potentials are truncated in the right panel. At this stage, the number of neurons is maximal (Anselmi et al., 2022). (C) Recording from the degenerating brain of a zooid approaching takeover (stage A5). Spontaneous activity is scarce and extracellular action potentials have a unitary waveform with little evidence of network activity. The inset shows an action potential on an expanded time base to illustrate the waveform. These results are representative of >100 recordings from >20 *B. schlosseri* clones.

As is typical of modular colonial species, *B. schlosseri* undergoes both sexual and asexual reproduction. On finding a suitable substrate, the tadpole larva metamorphoses and, through cycles of asexual reproduction (blastogenesis), gives rise to a sessile colony. The colony is composed of groups of individual zooids each about 2 mm in length arranged around a shared excurrent siphon to form a module (Fig. 1A). Every zooid is a filter-feeding individual with a brain and nervous system, heart, branchial sac and digestive system. Modules of multiple zooids are linked together by a common vascular network allowing the sharing of blood cells to preserve the clonal integrity of the colony.

Zooids undergo continual cycles of regression and renewal, during which the cells of the adult zooid die by apoptosis and are removed by circulating macrophage-like phagocytic cells (Lauzon et al., 1993; Ballarin et al., 2010). At the same time, a new generation develops from buds that mature into new adult zooids. This process has been termed takeover and it occurs synchronously in all the zooids in the colony (Lauzon et al., 1993). During maturation, all neural, somatic and germline lineages, plus the rudiment of the next generation of buds, are generated anew from adult stem cells, resulting in a colony of genetically identical individuals (Laird et al., 2005; Rinkevich et al., 2013; Rosental et al., 2018; Kowarsky et al., 2021). Under our culture conditions, takeover repeats every 7 days while the colonies themselves live for years. Therefore, at any time, a colony contains three overlapping blastogenic generations, adult zooids, primary buds and secondary buds.

Two excitable systems guide behavior in the *B. schlosseri* colony: the brain and nervous system of the individual zooids (Anselmi et al., 2022) and the electrically excitable, colony-wide vascular network (Mackie and Singla, 1983)*.* We found that each plays a prominent role during a different phase of the life cycle. The brain of a mature zooid is a cortical ring of neuron cell bodies surrounding a core of densely packed neurites where synaptic connections are made, an arrangement characteristic of many invertebrate nervous systems (Anselmi et al., 2022; Braun and Stach, 2019; Kowarsky et al., 2021; Zaniolo et al., 2002; Bullock and Horridge, 1965). Five pairs of major nerves exit the brain to innervate the siphons, branchial sac, body muscles, cilia and other organs (Burighel et al., 2001). The nervous system originates from stem/progenitor cells in the secondary bud and reaches maturity in the adult zooid (Kowarsky et al., 2021). The number of neurons increases to a maximum of approximately 900 cells 3 days after the siphons of the new zooid open and then declines during the second half of the zooid life cycle (Anselmi et al., 2022).

The vascular network extends throughout the colony, connecting with the heart and open circulation of every zooid and terminating in ampullae at the margin of the tunic (Mukai et al., 1978). Epithelial cells of the blood vessels are electrically excitable, generate action potentials both spontaneously and in response to mechanical stimulation, and are joined by gap junctions. Mackie and Singla (1983) found that epithelial action potentials participate in characteristic alarm contractions in response to stimulation.

We applied electrophysiology, video imaging and transcriptomics to study the relationship between the zooid nervous system and the excitable vascular network and to determine the role of each in organizing behavior. The cyclic nature of zooid renewal allowed us to repeat experiments in clone mates over multiple generations of the same organism. Our study revealed complex firing patterns in the zooid brain that underly sensorimotor behavior, some capacity for activity in sister zooids to synchronize, as well as patterned activity in the vascular network.

## MATERIALS AND METHODS

### Animal care

Colonies of *Botryllus schlosseri* (Pallas 1766) 0.5 to 2 years of age were obtained from the culture facility at the Hopkins Marine Station, Pacific Grove, CA, USA, placed in a lab incubator and maintained in filtered seawater at 20°C on a 11 h:13 h light:dark cycle. The medium was exchanged and the colonies were fed with rotifer culture every 4 days. For recording, a colony adherent to a glass coverslip and bathed in filtered seawater was mounted on a temperature-controlled microscope stage (20°C). Experiments were performed between 11:00 and 16:00 h. Colonies returned to the incubator after recording could be maintained in a healthy condition for several weeks, permitting repeated experiments on the same colony over numerous cycles of blastogenesis. All experiments conformed to the animal care standards of Stanford University.

### Staging colony development

The developmental stage of *B. schlosseri* colonies was determined using the staging convention described by Anselmi et al. (2022), adapted from Kowarsky et al. (2021). Zooids undergo repeated cycles of regression and renewal on a 7 day cycle. The time when the cells of the adult zooid are removed by circulating, macrophage-like cells (Lauzon et al., 1993; Ballarin et al., 2010) and the developing primary bud becomes the new adult is termed takeover. We take this as the starting point of the new cycle. Subsequent days in the cycle are termed A1–A6, the days leading up to the next takeover event. The nervous system begins to form in the secondary bud and continues to develop in the primary bud. Neurons continue to be added in the adult brain during days A1 and A2, and reach a maximum number on A3. Neurons are then lost progressively over the next 4 days leading to takeover. This cycle occurs synchronously in all the zooids in the colony.

### Electrophysiology

Extracellular recordings were made using polished glass pipettes (tip diameter 20–50 µm) filled with filtered seawater and fitted with an Ag:AgCl wire. The recording electrode was pressed to the tunic directly over the brain, the zooid body wall or over an ampulla of the vascular network using gentle suction. Voltage was recorded differentially between the pipette and an Ag:AgCl wire in the bath using AC-coupled preamplifiers (P55, Grass Instruments, Astro-Med, Warwick, RI, USA). Signals were amplified by a factor of 1 K or 10 K and filtered between 30 Hz and 1 KHz (Frequency Devices, Ottawa, IL, USA) before digitizing at 2 KHz using a DATAQ Instruments DI-1000 AD-converter and WinDaq software (Dataq Instruments, Akron, OH, USA). Digital records were analyzed with Igor Pro (WaveMetrics, Portland, OR, USA). A pneumatic microinjector (Picospritzer II, Parker Hannifin, Hollis, NH, USA) was used to present water-jet stimuli of variable strength and duration. Water jets were delivered from a glass micropipette and timed by a microprocessor with timing recorded by the AD converter.

### Video imaging

Colonies were viewed with a Wild stereo microscope fitted with a temperature-controlled stage (20°C) and an AmScope MD500 digital camera (Irvine, CA, USA). Illumination was directed from above or below. Images were collected using AmScope.com software at resolutions between 800/600 and 1280/1024 ppi and a frame rate between 0.5 and 12.5 frames s^−1^. Series of images were analyzed using Fiji (ImageJ) and Igor Pro (WaveMetrics) and changes in pixel intensity were measured in regions of interest (ROIs) positioned over the zooid, typically over the incurrent siphon, the margin of the zooid, or an ampulla of the vasculature. Contractions of zooids or ampullae resulted in an increase or decrease in reflected light intensity depending on placement of the light source, and were quantified either as the change in average pixel intensity or the standard deviation of pixel intensity in the ROI as a function of time.

### Transcriptomes and gene analysis

**Fig. 2.**
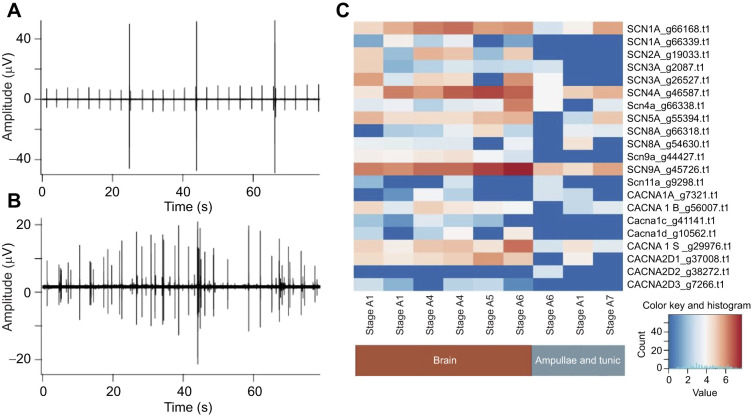
**The brain and vascular network both generate action potentials and both express genes homologous to those associated with voltage dependent sodium and calcium channels in vertebrate nervous systems.** Simultaneous recordings from an ampulla of the vascular network (A) and the mature brain of a nearby zooid (B) at stage A3. In this example, the ampulla exhibits rhythmic firing that is not seen in the neural record. Action potentials at the two sites were neither simultaneous nor phase-locked. (C) Expression patterns of putative homologous genes associated with human voltage-dependent sodium and calcium channels in the brain and ampullae of *B. schlosseri* (*x*-axis). Each gene is represented by a single row in the heatmap, where blue indicates low expression and red indicates high expression in log_2_cpm. The gene annotation is noted next to the *B. schlosseri* unique gene identifier (g). The columns indicate the tissues sampled (brain or ampulla) and the blastogenic stage. Although most of the candidate homologs are continually expressed in brain tissue throughout the neurodegeneration cycle, only a subset of the genes associated with sodium channels are expressed in the ampullae.

We used the protocol described in Anselmi et al. (2022) to dissect the tissues and prepare and sequence the libraries. The determination of genes was done as described in Kowarsky et al. (2021) and Anselmi et al. (2022). The heatmap presented in Fig. 2 was produced using R statistical software version 4.0.1 (https://www.r-project.org/).

## RESULTS

### Electrical activity in the *B. schlosseri* brain changes throughout the life of the zooid

Anselmi et al. (2022) studied changes in the *B. schlosseri* brain during development and found that the number of neurons in the brain first increases, reaching a maximum of ∼900 cells 3 days after the zooid opens its siphons, and then decreases gradually over the next 4 days as the zooid approaches takeover. They found that the decrease in neurons is correlated with diminishing response to mechanical stimulation.

We placed an external focal electrode against the tunic directly over the zooid brain in order to record neuronal action potentials (Fig. 1A). Fig. 1B shows spontaneous activity in the brain of a mature zooid when the number of neurons is at its peak (stage A3). The record is displayed at higher amplification to the right to illustrate a complex pattern of activity composed of multiple units that differ in amplitude. This is the kind of activity one expects of a robust nervous system containing numerous sensory, integrative and motor neurons. Spontaneous activity in the brain changes dramatically during the weekly life cycle. Fig. 1C shows a recording from the brain of a zooid near the end of its life (stage A5–A6). Action potentials are rare at this time, and they have a simple waveform characteristic of a single neuron. Multiunit firing patterns are lacking, which is consistent with the loss of neurons owing to programmed cell death, a phagocytic process whose time course was well documented by Anselmi et al. (2022).

### The electrical activity of the brain and ampullae are independent

Mackie and Singla (1983) and Mackie and Wyeth (2000) described electrical activity in ampullae of the vascular system of *B. schlosseri.* We used extracellular recording to determine whether action potentials in the ampullae and the brain are synchronized. Fig. 2 shows simultaneous recordings from an ampulla (panel A) and from the brain of a nearby mature zooid at stage A3 (panel B). In this example, the ampulla exhibits remarkable rhythmicity that is not seen in the neural recording. There is no indication of simultaneous or phase-locked activity at the two sites, which indicates that they act independently and that the vascular network itself has the properties of a pacemaker. This conclusion is supported by more than 50 experiments in several different clones. The three large action potentials in the ampulla record resemble potentials Mackie and Singla (1983) named ciliary arrest potentials.

To better understand the genes involved in the electrical excitability of adult zooids during the weekly developmental cycle, we looked for the expression of genes involved in membrane excitability and action potential generation at each stage of development. We identified 21 candidate genes homologous to human genes for voltage-dependent sodium and calcium ion channels. Except for the homolog to *CACNA2D2*, all candidate homologs are expressed in the brain throughout the zooid maturation/degeneration cycle without significant variation (Fig. 2C). Six of these genes are not expressed in ampullae, whereas four others are expressed in all the ampulla samples (Fig. 2C).

### The frequency of siphon contractions follows the rhythm of brain development and degeneration

**Fig. 3.**
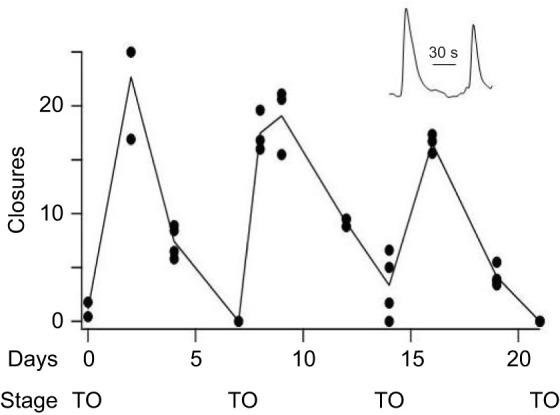
**Spontaneous siphon closure follows the rhythm of brain maturation and degeneration.** ROIs corresponding to the oral siphon were assigned to all six zooids in the same module. An example of two successive siphon closures is shown in the insert (closures are upward deflections). The number of siphon closures was counted in each zooid during 15 min video recording sessions spread over 21 days. Data were collected between the hours of 11:00 and 14:00 h every 2 to 3 days. The sum of closures in all the zooids is plotted in the graph (circles). In most cases two or more repeat measurements were made, separated by 20 min intervals. The line connects the means of repeat measurements. Between experiments the bathing medium was refreshed and the colony was returned to the incubator. There was no other stimulation. The video rate was 12 frames s^−1^ and siphon closures were scored if the reflected light intensity during the movement changed by >5%. The state of zooid development was determined from inspection of photomicrographs and the time of takeover (TO) is noted on the graph.

Anselmi et al. (2022) showed that the sensitivity of *B. schlosseri* to sensory stimulation changes over time in parallel with the maturation and degeneration of the zooid brain, possibly reflecting changes in the integration of sensory and motor pathways. We found that a similar conclusion applies to centrally generated behavior. One conspicuous zooid behavior is the periodic, but usually non-rhythmic, closing and reopening of the oral siphon, a movement coordinated by the nervous system. We used video imaging to monitor siphon behavior in a module composed of six zooids and counted the number of siphon closures in all six during 15-min observation windows over a period of 21 days, approximately three cycles of blastogenesis. The life stage of zooids at the time of measurement was determined from inspection of photomicrographs as described in the Materials and Methods. Fig. 3 shows that the frequency of closures follows a rhythm with a period of approximately 7 days. The time of each successive takeover is shown on the graph as an indicator of developmental time. The number of neurons in the brains of new zooids increases to a maximum of 780–900 in the first 4 days of the zooid's life and gradually declines to zero by the time of the next takeover (Anselmi et al., 2022). The rhythm of siphon activity follows approximately the same time course, peaking when the nervous system reaches maturity, declining during the second half of the life cycle and disappearing at takeover. This makes the point that oral siphon motility is generated by circuits in the brain and mediated by central motoneurons, an observation that allows us to use siphon activity as a proxy for brain development.

### The brain and vascular network take dominant roles in the control of behavior at different times during the zooid life cycle

**Fig. 4.**
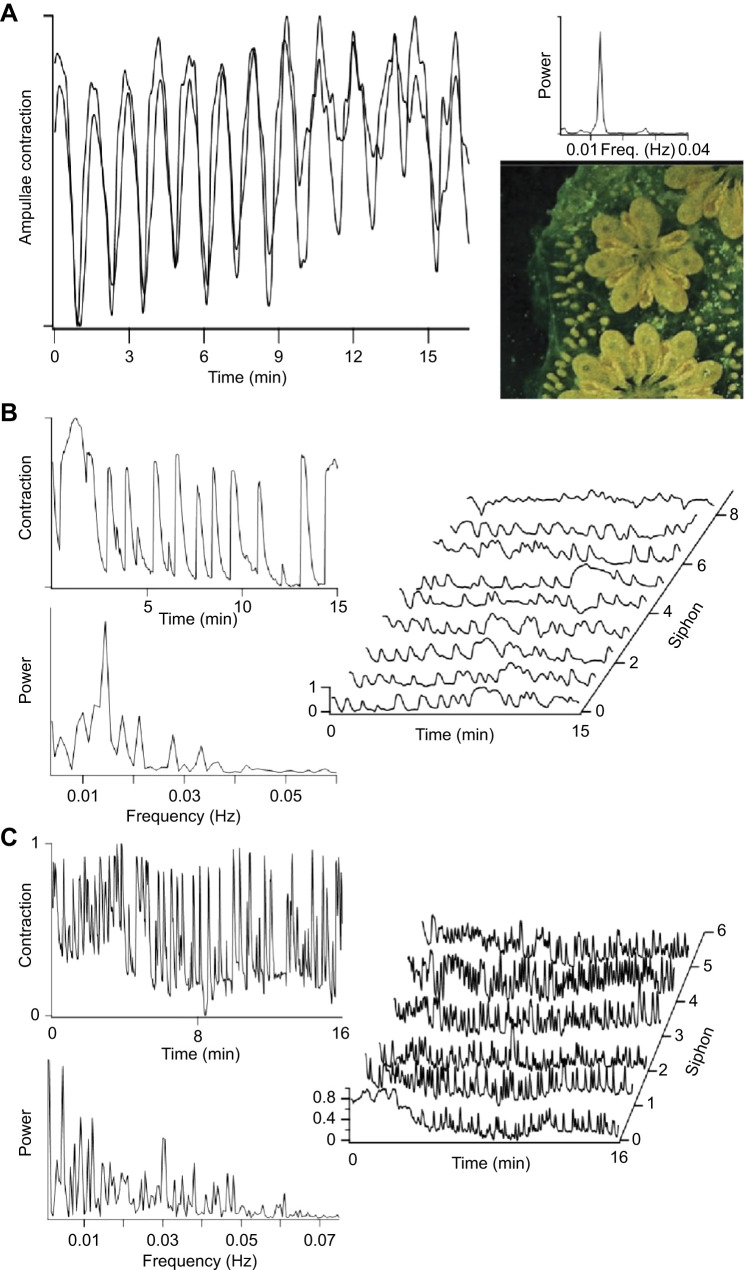
**Synchrony in the vascular network.** (A) Synchronous contraction in two ampullae spaced 5 mm apart in the same colony during a 17 min video recording. The periodogram of contractions (shown to the right) has a single peak at ∼0.013 Hz. The colony is in takeover and the photograph shows the swollen ampullae, degenerating zooids and developing primary buds characteristic of this developmental stage. (B) Movement recorded at the region of the oral siphon in a zooid nearing takeover (stage A4–A5). The periodogram of the movement also has a peak at ∼0.013 Hz indicating that the vascular network makes a strong contribution to timing the movement. The waterfall plot to the right shows contractions measured at all eight zooids in the module recorded over a 15-min period. The presence of both synchronous and asynchronous movements suggests contributions from both the vascular and nervous systems. (C) The same module imaged 4 days later, a time when the new zooids have matured and the neuron cell count is maximal (stage A3). Oral siphon contractions in one zooid are shown in the upper left panel. The periodogram (lower left panel) now has a broad frequency distribution with a much less obvious component at the frequency characteristic of the vascular network. The waterfall plot for six zooids shows a large amount of activity with little evidence of synchronous behavior.

As zooids approach takeover, the ampullae of the vascular network swell with cells, including phagocytes involved in the programmed cell death and removal of degenerating zooids (Voskoboynik et al., 2007; Corey et al., 2016; Rosental et al., 2018). During this time, the ampullae pulsate synchronously throughout the entire colony, a motility thought to involve microfilaments (De Santo and Dudley, 1969). Fig. 4A shows contractions in two ampullae located on different branches of the capillary network 5 mm apart. A periodogram of the behavior has a peak frequency of 0.013 Hz, a rhythm characteristic of all the colonies we examined (mean±s.d.=0.011±0.002 Hz, *n*=35). Synchronous contraction suggests that the timing relies on a single pacemaker, or group of tightly coupled pacemakers within the vascular network, with the epithelial cells of the blood vessels possibly acting as an excitable syncytium. Videos taken during takeover show that contractions of the ampullae extend passively to the tunic so that the entire colony pulsates.

The synchronous contraction of ampullae dominates movements recorded at the siphons in colonies approaching takeover (stage A4–A5). Movement in the region of the oral siphon of a single zooid is shown in Fig. 4B. The periodogram of the movement has a strong peak at ∼0.013 Hz, the characteristic frequency of vascular pulsations. Movements at other frequencies may be the result of muscle contractions following motoneuron activity in the brain of the aging zooid. The waterfall plot shows siphon movements recorded simultaneously from all eight zooids in this module. At this stage, some of the activity occurs in phase, although not all movements are shared. This again is consistent with the vascular network having a strong influence.

Siphon behavior in new, maturing zooids in the same colony is dramatically different 4 days later, after the nervous systems of the new zooids have matured (Fig. 4C, stage A3). The siphons are much more active, and their movements are more varied in timing, duration and amplitude. The periodogram of contractions plotted for one of the zooids in the figure now has a rich frequency distribution, and vascular contractions, although still present, no longer make a major contribution. The time course of siphon mobility for all six zooids in the module is shown in the waterfall plot. There is now little evidence of synchronous behavior. We conclude that siphon movements at this stage result from motoneuron activity in the brains of the individual zooids. Two points can be made. First, colony behavior is influenced by both excitable systems, brain and vascular network, with each playing a prominent role during a different part of the weekly life cycle. Second, the functional maturation of circuits in the brain responsible for siphon movements develops rapidly, during the approximately 3 days it takes for the number of neurons in the brain to reach a maximum. *Botryllus schlosseri* presents a rare case in which we know the time course over which all the circuits necessary for a defined, integrated motor act mature.

### Synchrony between zooids

**Fig. 5.**
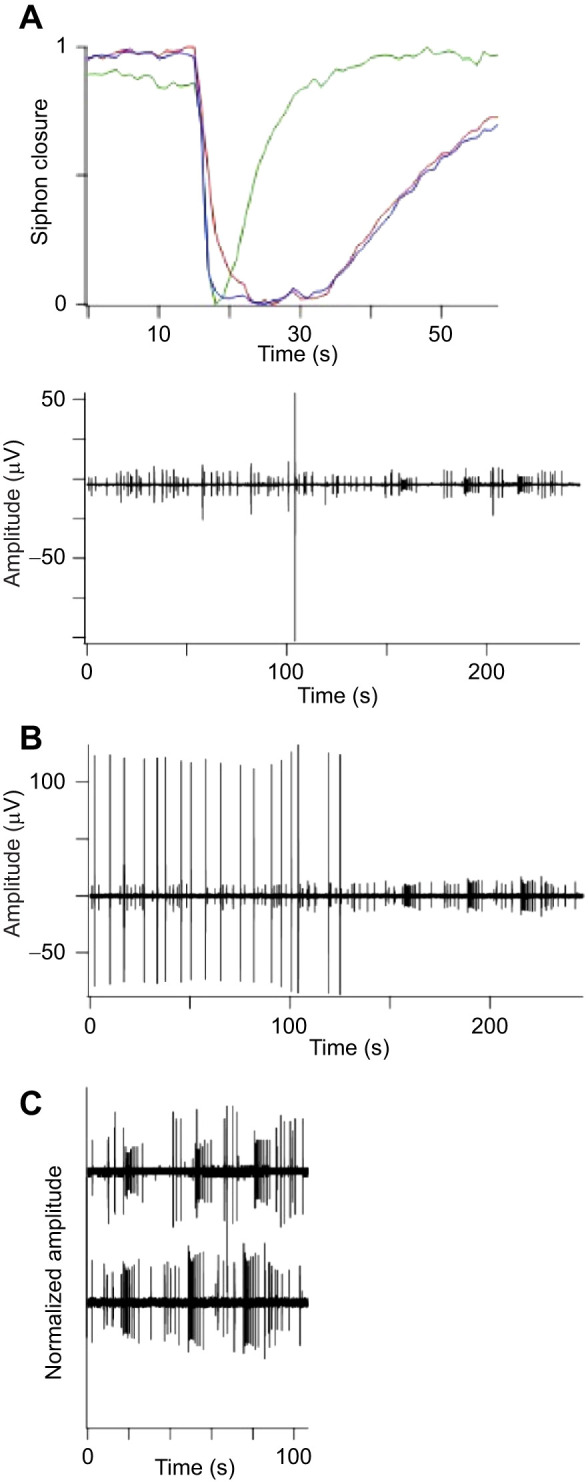
**Synchrony among zooids.** (A) Video recording showing synchronous oral siphon closure in three zooids in the same module in the absence of obvert stimulation (closure is shown downward). The colony was nearing takeover (stage A5). (B) Simultaneous electrical recordings from the brains of two mature zooids (stage A3) in a different module. The zooids were maximally distant from each other. Action potentials are seen to fire in bursts in the nervous systems of both zooids. (C) The second half of both records (*t*=150 to 250 s) are replotted at higher amplification to emphasize the firing patterns. Although the bursts are not strictly synchronous, there is strong similarity in timing and burst structure.

Motor behavior is sometimes synchronized among sister zooids. Fig. 5A shows nearly simultaneous siphon closures in three members of the same module in the absence of stimulation. We observed similar behavior in 25 experiments on 12 different *B. schlosseri* clones. Synchronous siphon closure can occur between as few as two zooids in a module, or it can involve all the zooids. It was most common at stage A3 and it sometimes occurred in clusters during long recordings. We have not identified conditions that favor synchronized movement, but its frequent occurrence suggests that a pathway exists to link zooid nervous systems, at least during some phase of the maturation/degeneration cycle. This could involve a neural pathway, although anatomical studies by others have not yet provided evidence of neural projections between zooids, leaving the issue unresolved. Synchrony could also result from mechanical coupling because the contraction of one zooid can exert a pull on its neighbors through the tunic that might activate mechanoreceptors and cause reflex contraction.

We recorded from the brains of sister zooids looking for phase-locked neural activity. An example of unstimulated activity in two zooids in the same module is shown in Fig. 5B. The largest spikes are not synchronous, but the timing and burst firing patterns of smaller action potentials are remarkably similar. Fig. 5C replots the second half of both records at higher amplification. Although the bursts are not strictly simultaneous, the similarity in burst structure is consistent with a neural pathway linking the two zooid brains. The significance of bursts and the types of neurons involved are not known and we have not yet identified specific behaviors associated with burst activity.

We used the same methods to search for neural pathways linking separate modules in a colony whose zooids are expected to be connected only by the vascular network. Individual bursts are not synchronous and no evidence was found for phase-locked activity in either the neural or behavioral records of these experiments (*n*>20). Our present conclusion is that zooids located in different modules are independent, at least with respect to control of the musculature. We are aware that this result may depend on the stage in the colony's life cycle, as well as specific behavioral states such as active feeding or the release of swimming larvae. It is worth noting that zooid modules are clearly linked by the processes responsible for synchronizing blastogenesis, processes thought to involve cells in the blood.

### Response to mechanical vibration

There are two types of ciliary mechanoreceptors in *B. schlosseri*: primary sensory cells and secondary sensory cells. Primary receptors send afferent axons to the brain, whereas the secondary sensory cells are innervated at their base by nerve fibers emanating from the brain. Both are sensitive to vibration. When stimulated, the primary sensory cells evoke contraction of the oral siphon, leading to oral siphon closure. Stimulation of secondary sensory cells evokes contraction of the excurrent siphon (Anselmi et al., 2022; Manni et al., 2018). The secondary sensory cells also control the flow of seawater into the organism and can drive what is called the ‘squirting’ reaction, a rapid contraction of body muscle contraction used to eject dangerous particles during filtration (Manni et al., 2018).

**Fig. 6.**
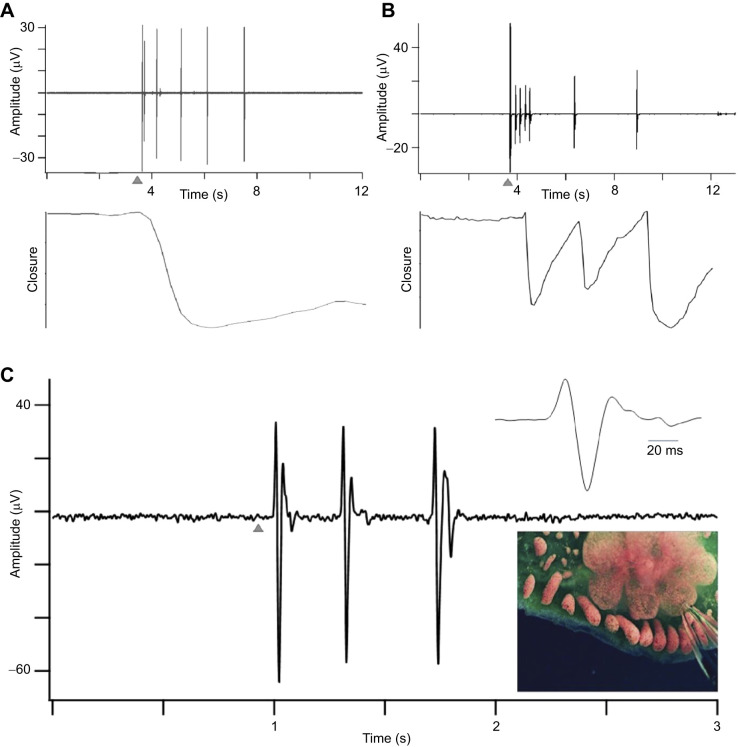
**Response to mechanical stimulation.** (A) Brain activity and siphon closure were recorded simultaneously. A single sharp tap applied to the isolation table at the arrow elicited a burst of action potentials beginning before the onset of contraction and lasting beyond its peak. Siphon closure is shown as a downward deflection. (B) The same experiment in an adjacent module in the same colony. In this example, the tap (arrow) resulted in multiple bursts of action potentials lasting a total of ∼9 s and resulting in three siphon closures. (C) Repeat of the experiment in a developing zooid before the siphons have opened (early-stage A). A single tap (arrow) elicited only a short burst of three action potentials at the site of the maturing brain. The insert shows one of these on an expanded time scale. The response is substantially different from the response in the mature nervous system, lacking the more elaborate firing patterns that include contributions from multiple units.

*Botryllus schlosseri* is acutely sensitive to physical disturbance, responding with vigorous contractions of body musculature and siphons to mechanical stimulation of the abundant mechanoreceptors (Anselmi et al., 2022; Kowarsky et al., 2021; see Mackie et al., 2006 for similar findings on a related species). The examples in Fig. 6A,B show responses in the brains of two zooids from different colonies, along with video records of oral siphon closure in response to a single tap applied to the vibration isolation table. In both cases, the tap elicited a prolonged burst of action potentials beginning before the contraction and lasting for 5 to 9 s. In Fig. 6B, the tap led to an initial burst and contraction, followed with delays of approximately 2 s by two additional bursts. Recurrent activity like this suggests a reverberating neural network involving integrative circuits in the brain.

We applied the same stimulus early in brain development before the siphons of new zooids had opened (Fig. 6C), a time when there is little spontaneous neural activity. The mechanical stimulus elicited three action potentials but did not result in muscle contraction, a much less robust response than in mature zooids. This suggests that sensory inputs responsible for reflex behavior develop early in brain development before the sensory integration and associated motor circuits necessary for sustained burst firing and a muscular response have formed. It appears that neural development proceeds in stages, with functional connections of sensory neurons to central neurons preceding the formation of more extensive neural circuits governing coordinated behavior.

### Response to focal stimulation

**Fig. 7.**
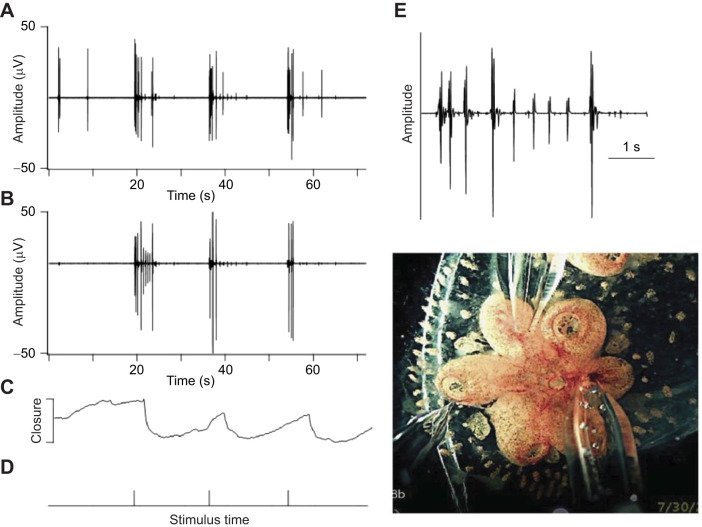
**Neuronal and behavioral responses to repeated focal stimulation.** (A) A 10 ms water jet from a glass micropipette was used to stimulate a module of zooids while recording from the brains of two of them (A and B). The water jet was pointed at a third zooid (leftmost pipette in the photo). Stimulus timing is shown in D and the resulting closure of one of the oral siphons is shown in C. Each stimulus elicits a sustained burst of action potentials that begins before the siphon contraction. The burst pattern at the beginning of the response to the first stimulus in B is shown on an expanded time scale in E to illustrate the complicated burst structure.

Anselmi et al. (2022) described reflex behaviors resulting from focal stimulation of the siphon with miniature water jets. We used a similar method to examine the effect of mechanical stimulation on neural activity. In Fig. 7, a glass micropipette positioned close to one zooid was used to deliver a 10 ms water jet while recording from the brains of two other zooids in the same module, distant from the site of stimulation. Three pulses were applied, and each caused a single oral siphon closure (Fig. 7C,D). The neural responses in sister zooids are shown in Fig. 7A,B. The firing patterns and the timing of activity in the two zooids were similar even though the stimulus was directed at a distant individual. Neural activity following the first stimulus in one of the zooids is shown on an expanded time scale in Fig. 7E. The latency between the onset of the stimulus and the beginning of the neural response was 90–110 ms and the response consisted of multiple bursts spaced between 250 and 800 ms apart. This complicated firing pattern shows that rather than a simple reflex, the stimulus elicits an integrated response in circuits involving multiple neurons. The significance of the prolonged bursting pattern is not known, but it suggests that the stimulus may activate pathways affecting several different behaviors, for example, those leading to ciliary arrest as shown by Arkett (1987) in a related tunicate and by Mackie and Singla (1983) in *B. schlosseri*.

## DISCUSSION

The regular cycle of blastogenesis in *B. schlosseri* allowed us to study nervous system function, neurogenesis and neurodegeneration repeatedly in the same colony. This makes *B. schlosseri* a very attractive model for the study of the physiology of the nervous system and its role in orchestrating behavior in a colonial organism at the base of the vertebrate evolutionary tree. One of our goals was to examine the degree to which the behavior of individuals in the colony is coordinated. A conspicuous feature of *B. schlosseri* colonies is the weekly cycle of zooid development and degeneration, something that follows the same time course in every individual. This remarkable synchronization involves well-timed sequences of stem cell activation, apoptosis and activation of phagocytic macrophage-like cells. It is not yet known how these events are coordinated, although it clearly involves signals shared via the vascular network because when vascular connections are cut, developmental synchrony is lost (Watanabe, 1953).

The colony-wide vascular network also exhibits synchronous behavior. Ampullae contract simultaneously throughout the colony and this is particularly apparent during takeover. Simultaneous contraction suggests that a vasculature pacemaker is involved in timing the behavior. Mackie and Singla (1983) showed that epithelial cells of the ampullae generate action potentials and suggested that epithelial conduction involving gap junctions may be responsible for synchronizing the network. Ampullae are known to express voltage-dependent Na^+^ and Ca^2+^ channels and gap proteins (Voskoboynik et al., 2013). If the electrical resistance of gap junctions is low enough and the coupling coefficient high enough, the transmembrane voltage of all the coupled cells will be nearly the same. In this case, a slow action potential or a pacemaker potential recorded at an ampulla is a network potential that will occur in all the ampullae at the same time. This would mean that the vascular network will act as an electrical syncytium, similar to astrocyte networks and certain neurons in the mammalian central nervous system (Bennett and Zukin, 2004; Ma et al., 2016). We recorded from pairs of ampullae but did not observe this kind of behavior. What we did find is that the rhythmic dilation and constriction in the colony wide vascular network is both very slow (∼0.01 Hz) and synchronous.

It is difficult to envision a mechanism based on rapid action potentials propagating in an electrically coupled network that can explain the slow ampullar rhythm. Gap junctions and membrane ion channels may be involved at some level, but we note that if a slow oscillation in membrane potential occurs, our extracellular recording method would not be able to resolve it. Similar very slow contractions have long been known in mammalian capillary beds, where they are termed vasomotion (Aalkjaer and Nilsson, 2005). This behavior is not fully understood, but one current idea is that it results from a form of cytoplasmic Ca^2+^ signaling involving intracellular Ca^2+^ release, Ca^2+^ influx and refilling of intracellular Ca^2+^ stores. It will be interesting to examine this idea in *B. schlosseri*.

Contraction of the oral siphon is a readily observed behavior that is controlled by the nervous system and follows the weekly rhythm of brain development and degeneration. This means that the neural circuits responsible for the siphon rhythm assemble over the first 3 days of zooid development and then become progressively less active during the second half of the cycle. In many of our experiments, the timing of siphon movements in sister zooids seemed to be independent, presumably addressing the needs of the individual. However, we observed simultaneous siphon behavior on numerous occasions and electrophysiology sometimes revealed remarkably similar burst firing patterns in sister zooids. Both could result from direct synaptic connections linking zooid nervous systems, although anatomical studies have not yet provided evidence for pathways linking zooid nervous systems (Anselmi et al., 2022; Burighel et al., 2001; Zaniolo et al., 2002). There is a caveat, however: brain activity changes with the zooid life cycle. This raises the possibility that synaptic connections supporting synchrony might appear transiently. A potential site for such a connection is the region of the excurrent siphon, where axons from all the zooids converge, potentially allowing communication between neuron terminals (Mackie and Wyeth, 2000). This merits further study giving careful attention to developmental time. A second possibility, also unresolved, is that mechanical coupling through movements imparted to the tunic stimulate mechanoreceptors to activate reflex responses in adjacent zooids.

Some details about the functional organization of the *B. schlosseri* brain are emerging. Anselmi et al. (2022) demonstrated that mechanical stimulation activates different behavioral responses depending on the type of receptor activated. This implies that there are several discrete sensory motor circuits. Our work shows that multiple neuron subtypes contribute in orchestrating muscular activity both in the absence of stimulation and in response to vibratory and water jet stimuli. Similarly, Arkett (1987) showed that specific, identified neurons in the brain of a related species control ciliary arrest in the branchial basket. The *B. schlosseri* nervous system, with its rapid turnover in response to still unidentified developmental cues, has the potential to provide new insights into the assembly of specific neural pathways during development.
